# Finite element modeling of human thorax for electrical bioimpedance based monitoring of pulmonary fluid accumulation

**DOI:** 10.2478/joeb-2026-0002

**Published:** 2026-03-15

**Authors:** Frijia Mortuza, Md. Shahriar Kabir, Md. Zaman Molla, Md. Ibrahim Al Imran, Muhammad Abdul Kadir

**Affiliations:** Department of Biomedical Physics and Technology, University of Dhaka, Dhaka, 1000, Bangladesh; Department of Sciences, BGMEA University of Fashion and Technology, Dhaka, 1230, Bangladesh; Department of Electrical and Electronic Engineering, Bangladesh Army University of Science and Technology, Saidpur, Bangladesh; Division of Physics, Department of Arts and Sciences, Ahsanullah University of Science and Technology, Dhaka, 1208, Bangladesh

**Keywords:** Bioimpedance, lung fluid, pulmonary edema, FEM modeling, segmentation

## Abstract

Monitoring fluid accumulation in the lungs is critical in conditions such as pulmonary edema and pneumonia. Current diagnostic modalities, including auscultation, chest X-ray, computed tomography, magnetic resonance imaging, and ultrasonography, either involve ionizing radiation or are not suitable for continuous long-term monitoring. This study investigated the feasibility of a non-invasive, non-ionizing electrical impedance–based approach for continuous assessment of pulmonary fluid accumulation using computational modeling. Firstly, CT images of human subjects were used to build a simplified thorax model. Different parts of human thorax including airways, left and right lungs, and soft tissue were segmented using a segmentation software Materialise Mimics^®^ and imported into COMSOL Multiphysics^®^ for finite element analysis. Tetrapolar transfer impedance was computed at multiple vertical electrode positions under baseline (air-filled lung) and fluid-accumulation conditions. The results demonstrated a measurable reduction in impedance in the presence of fluid, particularly at electrode levels corresponding to the fluid-filled lower lobes. A linear relationship between impedance and fluid volume was observed (R^2^ = 0.9972 for the left lung and R^2^ = 0.9998 for the right lung), with sensitivities of −466.74 mΩ/100 mL and −754.75 mΩ/100 mL, respectively. For clinically relevant fluid accumulations (≥300 mL), the predicted impedance change exceeded 2 Ω, indicating practical detectability. Frequency-domain analysis (5–1000 kHz) further demonstrated consistent impedance contrast across the investigated range. These findings suggest that tetrapolar electrical impedance measurements have the potential for continuous monitoring of pulmonary fluid changes and provide a foundation for future experimental validation in human subjects.

## Introduction

Pulmonary edema is a serious medical condition characterized by the abnormal accumulation of fluid in the lung tissue and alveolar spaces. This fluid buildup impairs gas exchange, often resulting in respiratory distress, hypoxia, and in severe cases, respiratory failure [1, 2]. Pulmonary edema can be broadly classified into cardiogenic and non-cardiogenic types, with common causes including congestive heart failure, renal insufficiency, acute respiratory distress syndrome (ARDS), infections, and certain medications [3, 4]. The clinical burden of pulmonary edema is significant. In patients with acute heart failure with reduced ejection fraction, the prevalence of cardiogenic pulmonary edema has been reported in the range of approximately 75–85% in contemporary clinical studies [5]. The condition is associated with significant in-hospital mortality, with rates reported between 3.8% and 36.5% across varied clinical settings, reflecting substantial clinical burden despite advances in heart failure management [6]. Given its high morbidity and mortality, timely diagnosis and effective monitoring of changes in pulmonary fluid accumulation are critical to improving patient outcomes. Beyond initial diagnosis, continuous monitoring of lung fluid changes is essential for assessing disease progression, evaluating treatment efficacy, and tailoring individualized therapeutic interventions.

Traditionally, chest radiography has been the most commonly used imaging modality for detecting pulmonary edema due to its accessibility and ease of use. More recently, lung ultrasound has gained traction, particularly in intensive care settings, owing to its high sensitivity in detecting interstitial fluid accumulation through B-lines [7]. Advanced imaging modalities such as computed tomography (CT), magnetic resonance imaging (MRI), positron emission tomography (PET), and single-photon emission computed tomography (SPECT) offer higher resolution and functional insights, but they come with notable drawbacks including radiation exposure (X-ray, CT), high costs (MRI, PET), limited availability, and lack of feasibility for repeated or continuous monitoring [8].

Moreover, physical examination and pulmonary function tests, though routinely performed, lack the sensitivity and specificity needed for reliable detection of early or subtle changes in lung fluid content. A critical gap remains in the ability to continuously and noninvasively monitor changes in pulmonary fluid status in real time, particularly in high-risk or critically ill patients [9].

Although conventional imaging techniques can detect pulmonary edema, their limitations, especially in continuous and radiation free monitoring, necessitate the exploration of alternative methods. In this context, electrical bioimpedance presents a promising alternative. Bioimpedance measures the frequency-dependent opposition of biological tissues to electrical current, which is influenced by the tissue’s dielectric properties, such as conductivity and permittivity [10]. Importantly, this technique is noninvasive, non-ionizing, and suitable for repeated or continuous monitoring, and has been successfully applied in diverse clinical applications including body composition analysis, tissue hydration assessment, cancer detection, and impedance cardiography [11].

Research has also demonstrated the feasibility of using bioimpedance to probe deep organs such as the lungs and stomach through surface electrodes [12,13,14]. Dielectric properties of lung tissue is a function of air content, and electrical conductivity as well as the relative permittivity decreases with increased air filling of the lungs [15]. Since fluid accumulation alters dielectric properties in lung tissue, changes in bioimpedance can be used as indirect markers of pulmonary edema. In particular, electrical impedance tomography (EIT)—which reconstructs cross-sectional images of internal conductivity using electrode arrays—has been explored for visualizing lung fluid dynamics in pulmonary edema [16, 17]. Additionally, impedance spectroscopy studies on simplified torso models have shown that reliable monitoring may be achievable with as few as 8–10 electrodes [18].

Despite these advances, there remains a need for a realistic, anatomically informed modeling approach to further validate and optimize bioimpedance techniques for changes in pulmonary fluid using a minimal number of electrodes. Finite element modeling (FEM) provides a powerful computational tool to simulate the electrical behavior of complex biological structures. By incorporating detailed anatomical data, FEM can be used to investigate the normal and localized diseased conditions [19]. Before conducting feasibility of bioimpedance based monitoring of lung fluid directly on human subjects, a finite element model-based study on a realistic model of the human thorax can be very useful in reducing risks and costs associated with physical prototypes and real-world trials.

The aim of this research was to develop a simplified finite element model of the human thorax and to study the utility of the electrical bioimpedance method in the monitoring of fluid accumulation in lungs. This study shows the 3D segmentation of left and right lungs and other body parts of the human thorax from CT images. Using tetrapolar electrodes placed on the chest surface, simulations were conducted using COMSOL Multiphysics^®^ to evaluate impedance variations in normal versus edematous lungs. The aim is to assess the utility of bioimpedance techniques to monitor the changes in fluid accumulation in lungs. The findings aim to support the development of a practical, radiation free and effective bioimpedance based tool for monitoring lung fluids.

## Materials and methods

### Image segmentation and 3D anatomical model generation

Image segmentation is a technique in medical image analysis, involving the division of digital images into multiple regions that correspond to different anatomical structures or tissue types. These regions, or segments, are composed of voxels sharing similar intensity characteristics, which are typically associated with tissue density or contrast in imaging modalities such as computed tomography (CT). In this study, segmentation was performed to extract and reconstruct the anatomical structures of the human thorax, specifically the left lung, right lung, airways, and surrounding soft tissues. The goal was to create a simplified, yet anatomically representative, 3D model suitable for bioimpedance-based simulation studies.

**Fig.1:**
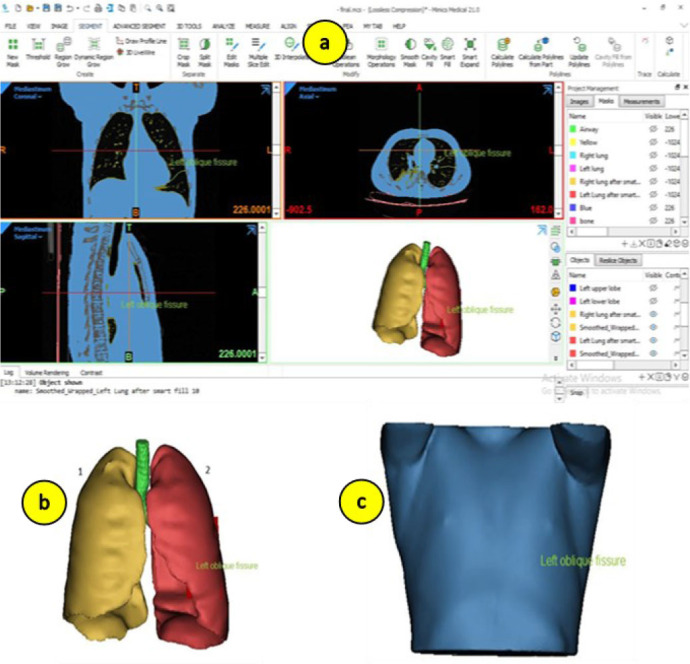
(a) User interface for image segmentation software, (b) segmented left and right lungs with airways, (c) 3D anatomical model of human thorax.

The de-identified CT data used for segmentation were sourced from an open-access repository, the Harvard Dataverse (Subject No. 1016), consisting of 518 slices of the thoracic region [20]. Prior to segmentation, the CT images were imported into Materialise Mimics^®^ (v21.0), a widely used medical image processing software approved for clinical and research applications [21]. Mimics offers a comprehensive suite of tools for thresholding, region growing, manual editing, and 3D reconstruction. Figure 1a shows the graphical user interface of the Materialise Mimics^®^ software.

Tissue segmentation was performed using intensity-based thresholding according to Hounsfield Unit (HU) values. Low HU thresholds (approximately −1000 HU) were applied to isolate the major airways. The lung parenchyma was segmented using a threshold range of −900 to −300 HU, while higher HU values were used to segment the surrounding region as a single tissue, merging soft tissues and bony structures. For simplicity, the volume around the lungs was considered equivalent to soft tissue.

Region growing techniques were employed to expand the masks from selected seed points within each region of interest, helping to capture the full extent of each anatomical structure. Manual editing tools, such as brush and lasso functions, were then used to correct any inaccuracies. Once segmentation was complete across all slices, the individual 2D masks were compiled into a 3D volume. Figure 1b illustrates the segmented and 3D-reconstructed left and right lungs. The generated 3D geometry of the thorax comprised the left lung, right lung, major airways, and surrounding soft tissue.

For the purposes of this study, each segmented region was considered a piecewise homogeneous domain, simplifying the model while preserving the essential anatomical features relevant to impedance analysis. The lungs and major airways were modeled as distinct internal volumes, while the surrounding space was assumed to be homogeneous soft tissue, representing a combination of muscle, fat and the resulting 3D anatomical model was exported in STL format for use in further simulations. Figure 1c shows the 3D anatomical model of the human thorax containing lungs and soft tissue.

### Finite element modeling and bioimpedance simulation

**Fig.2:**
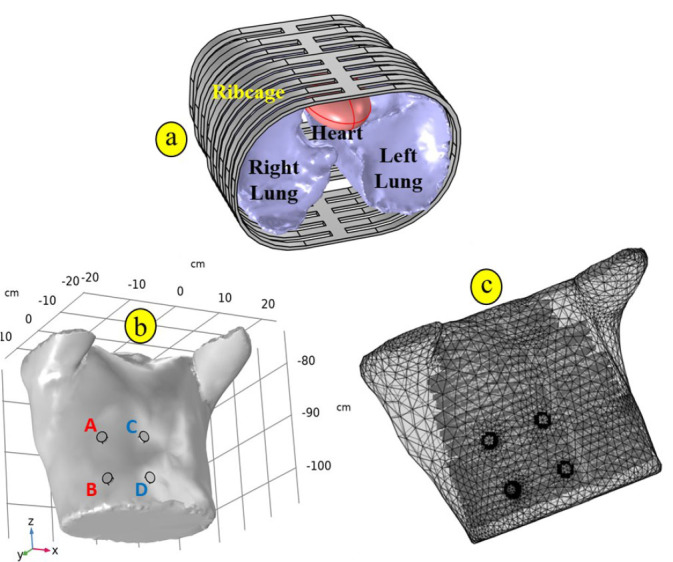
(a) Three-dimensional geometry of the ribcage enclosing the lungs and heart, with the heart located between the left and right lungs, (b) 3D geometrical model imported to FEM software showing electrodes placed on the surface of the thorax, (c) finite element mesh representation of the thoracic model.

The segmented anatomical model of the human thorax was imported into the COMSOL Multiphysics^®^ software platform to perform finite element based bioimpedance simulations. COMSOL offers a robust environment for simulating electrical, mechanical, and multiphysics phenomena and is particularly well-suited for modeling complex geometries derived from medical imaging data. For better representation of the human thorax a ribcage consisting of 12 pairs of ribs and an ellipsoid heart were modeled using COMSOL’s inbuilt geometry operations, as shown in figure 2a. The ribcage was embedded within the soft tissues, surrounding the lungs whereas the heart was placed in between two lungs.

To simulate bioimpedance measurements, four cylindrical electrodes made of Steel AISI-4340 (electrical conductivity = 4×10^6^ S/m, relative permittivity = 1) were positioned on the surface of the thorax model as shown in Figure 2b. The diameter of each electrode was 1 cm, and their placements were defined according to a tetrapolar configuration, where the electrodes were placed on the corner of a square. The center to center distance between two adjacent electrodes were considered as electrode separation. This configuration is commonly employed in bioimpedance measurements to reduce the influence of electrode contact impedance on voltage readings.

The meshing of the geometry was performed using COMSOL’s built-in mesh generator. Physics controlled mesh with ‘Normal’ element size was generated to accurately capture the geometry of the anatomical domains of the thorax. Figure 2c shows the 3D mesh representation of the thoracic model used in this study. The volume of the generated 3D thorax was 23.4 L while the volume of the inflated lungs was 4.84 L, indicating that the segmented model captured realistic proportions of anatomical structures.

The simulation was conducted using the AC/DC module of COMSOL Multiphysics^®^ in the frequency domain, with an excitation frequency of 10 kHz, which falls within the range typically used in tissue bioimpedance studies. A frequency of 10 kHz was selected as it lies within the extracellular-dominated β-dispersion region, maximizing sensitivity to interstitial conductivity changes while minimizing electrode polarization effects. The electric potential distribution within the thoracic volume conductor was governed by equation (1):

(1) $$\nabla \cdot \left(\sigma \nabla V\right)=0$$

where σ denotes tissue electrical conductivity and V the electrical potential.

Since the electrical conductivity of air is several orders of magnitude lower than that of biological tissues, current flow into the surrounding air is negligible. The external boundary of the thorax was modeled as electrically insulating (***J*** · ***n*** = 0). At the interfaces between internal domains, the continuity condition was enforced to ensure the conservation of current and electric potential across tissue boundaries. Additionally, the inner surfaces of the airways were considered electrically insulated, reflecting the non-conductive nature of air within the tracheobronchial tree.

For the purposes of simplifying the computational model while preserving physiological relevance, each tissue domain within the thorax was assumed to be homogeneous and isotropic. That is, electrical conductivity and permittivity were spatially uniform within each tissue type, but different for different types of tissues modelled. The lung tissue (inflated) was assigned a conductivity of 0.0932 S/m and a relative permittivity of 17,174. The surrounding soft tissue (primarily muscle and adipose tissue) was modeled with a conductivity of 0.18233 S/m and a relative permittivity of 13,497. The ribs were assigned a conductivity of 0.0515 S/m and a relative permittivity of 1,089 representing average of cancellous and cortical bones, whereas the heart tissue was modeled with a conductivity of 0.1542 S/m and a relative permittivity of 70,054. All dielectric properties were extracted at 10 kHz from published databases [22]. Since the study focused on conductive changes associated with pulmonary fluid accumulation, tissues were modeled using real-valued electrical conductivity (σ) and relative permittivity (ε_r_).

To compute bioimpedance using the tetrapolar electrode arrangement, an alternating current I_0_ (1 mA, peak amplitude) was injected through the electrode pair A–B (see figure 2b). Current injection was implemented using a Terminal boundary condition on the source electrode (A), enforcing:

(2) $$\int_{S}J\cdot n\;\mathrm{dS}=I_{0}$$

where **J** is current density, **n** is the outward normal vector, and I_0_ is the total injected current (1mA). The return electrode (B) was defined as electrical ground (0 V). This formulation ensures a fixed total current while allowing spatial redistribution of current density across the electrode surface. The corresponding reciprocal current was applied through the electrodes C–D (see figure 2b). Transfer impedance was computed using the reciprocity sensitivity formulation, which is equivalent to the conventional tetrapolar measurement in linear conductive media but provides direct access to spatial impedance sensitivity distributions. If **J**_AB_, **J**_CD_, are the current density vectors at a point within the thoracic volume for injection of current I_0_ through the electrode pairs (A-B) and (C-D), respectively then the volume impedance density at that point is given by equation (3) [23].

(3) $$\text{Volume}\;\text{impedance}\;\text{density},\;\text{z}=\frac{1}{\sigma }\frac{J_{\mathrm{AB}}\cdot J_{\mathrm{CD}}}{I^{2}}$$

Volume impedance density in tetrapolar transfer impedance measurements is the sensitivity multiplied with the local resistivity and refers to how responsive the measured impedance is to changes in tissue conductivity at different locations within the volume between and around the electrodes. It indicates how much a small change in tissue (like fluid accumulation) at a certain point affects the measured impedance.

The magnitude of the total thoracic transfer impedance (Z), referred to simply as *impedance* throughout the article, was then obtained using equation (4) [23].

(4) $$Z=\int_{v}\frac{1}{\sigma }\frac{J_{\mathrm{AB}}\cdot J_{\mathrm{CD}}}{I^{2}}\mathrm{dv}$$

Here σ is the local conductivity and the integration is over the whole volume of the thorax.

Electrodes were modeled as high-conductivity metallic domains (σ_electrode_ = 4×10^6^ S/m), which is several orders of magnitude higher than the conductivity of thoracic tissue compartments. In the tetrapolar configuration, the sensing electrodes draw negligible current; therefore, electrode–tissue interface impedance does not significantly contribute to the measured transfer impedance. Furthermore, at the operating frequency of 10 kHz, electrode polarization effects are minimal. Therefore, explicit electrode–skin contact impedance was not included in this model. The frequency-domain problem was solved using an iterative linear solver (BiCGStab) with a relative residual tolerance of 10^−3^.

A mesh convergence analysis was conducted using four refinement levels (coarser, coarse, normal, and fine) by placing electrodes on the lower right portion (figure 2c). The computed transfer impedance values were 15.027 Ω (coarser), 15.079 Ω (coarse), 15.082 Ω (normal), and 15.095 Ω (fine). The maximum deviation relative to the fine mesh was less than 0.5%, while the difference between the normal and fine meshes was below 0.1%. These results demonstrate numerical stability and mesh independence of the solution for the normal and finer meshes. The normal mesh was therefore adopted for all subsequent simulations. There were 126466 tetrahedral, 24630 triangular, 4196 edge and 566 vertex elements in the mesh.

**Fig.3:**
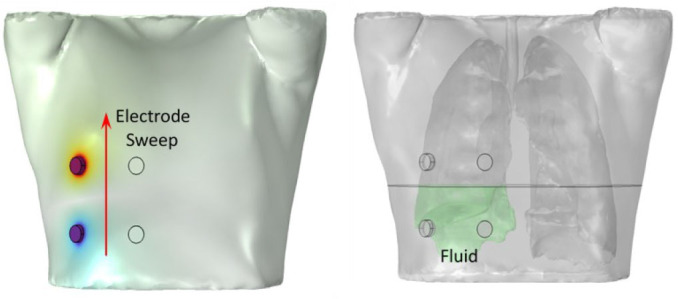
Tetrapolar electrode configuration and sweeping of electrode array along different vertical levels of the thorax (left). Lower portion of the right lung modeled as fluid buildup by assigning dielectric properties of body fluid (right).

### Study of fluid accumulation in lungs

The primary objective of this study was to evaluate the sensitivity of electrical impedance measurements to detect changes in fluid accumulation in the lungs, as typically observed in conditions such as pulmonary edema. These conditions are characterized by the retention of fluid within specific regions of the lung parenchyma, which can obstruct air exchange in the alveoli and alter the electrical properties of the lung tissue. Previous clinical studies have established that pulmonary fluid accumulation tends to initiate in the lower lobes of the lungs, especially when the patient is in an upright position, due to the influence of gravity [24].

To simulate this physiological condition, a modified version of the segmented thorax model was developed. In this version, a localized region in the lower part of the right/left lung was designated to represent fluid accumulation as shown in figure 3. In pulmonary edema, Extravascular lung water (EVLW) commonly increases to >10–15 mL/kg, representing an additional 0.3–1.0 L above baseline [25]. Therefore, the volume of the simulated fluid buildup was arbitrarily chosen to be 0.670 L. This edematous region was assigned the electrical conductivity σ = 1.5 S/m and relative permittivity ε_r_ = 98 at 10 kHz, to approximate interstitial/alveolar fluid [22]. These values reflect the high ionic content of interstitial and alveolar fluid, which significantly differ from the properties of normal lung tissue.

To assess how this localized change in tissue properties affects bioimpedance measurements, simulations were conducted by sweeping the electrode configuration along the vertical axis of the thorax (see figure 3). A tetrapolar electrode configuration was used, with electrodes placed on the surface of the chest. The position of the electrode array in which the bottom two electrodes align with the lower margin of the lung was considered as the reference position. The electrode array was systematically shifted vertically upwards in increments of 2 cm to scan the thoracic volume at different vertical positions. The term “vertical position” refers to the location of the electrode array on the anterior surface of the thorax, while the electrode separation was kept constant at 8 cm for all simulations.

At each vertical position, the electrical impedance (Z) was computed using the same simulation protocol described earlier, in both normal (without fluid) and pathological (with fluid) conditions. The simulation was carried out separately for the left and right lungs by placing electrode on the left and right side of the chest to compare lateral differences.

To investigate the effect of fluid volume on impedance measurements, simulations were performed in which the amount of fluid accumulation in the lower lobes of the left and right lungs was varied. For each fluid volume, the corresponding impedance was computed to assess the sensitivity of the measurement to changes in fluid content.

To examine the frequency dependence of the impedance response, simulations were performed at multiple excitation frequencies: 5, 10, 50, 100, 200, 500, and 1000 kHz. At each excitation frequency, real-valued conductivity σ(ω) and permittivity ε(ω) were assigned based on literature values [22], as summarized in Table S1 (Supplementary Material). The simulations were conducted in the frequency domain and the transfer impedance was computed for each frequency under both baseline (no fluid) and fluid-accumulation conditions.

### Ethical approval

No new human or animal data were collected for this study.

## Results

**Fig.4:**
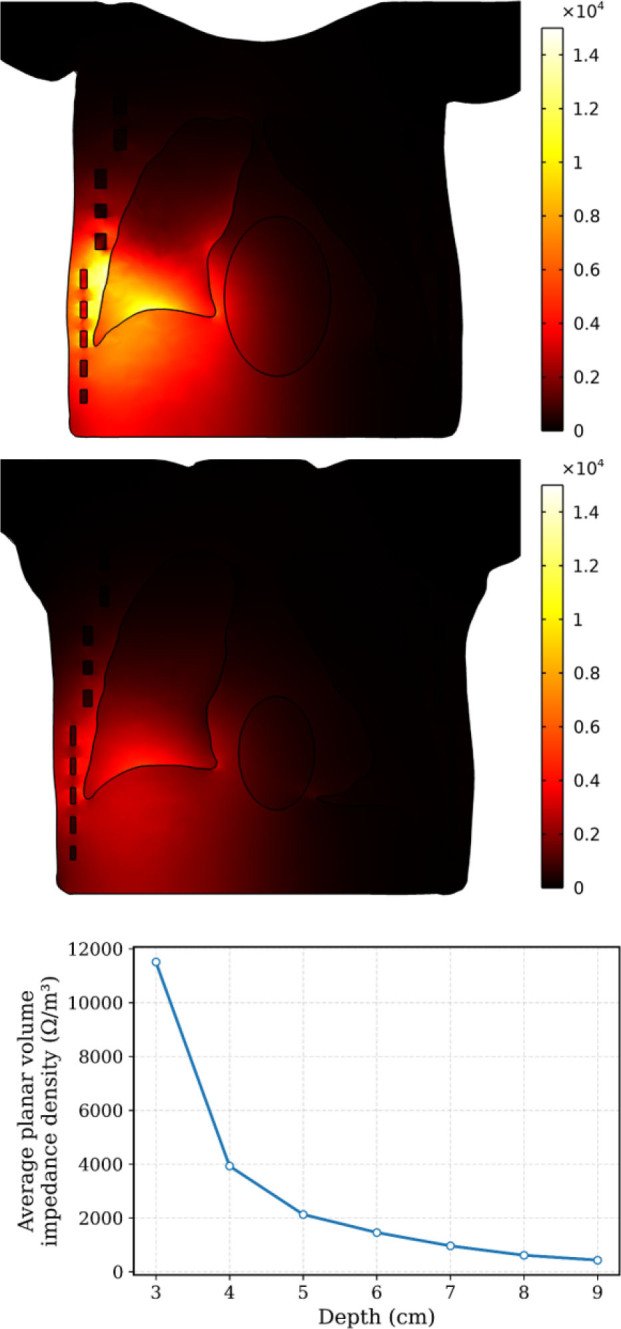
Spatial sensitivity distribution along the planes 6*cm* (top) and 8*cm* (bottom) away from the electrode plane (thorax surface). The unit of volume impedance density in the color bar is Ω/m^3^. Average planar volume impedance density variation with depth from the surface of the thorax (bottom).

Volume impedance density in impedance measurements quantifies how strongly a small change in tissue properties at a specific location influences the measured impedance. Using finite element method (FEM) simulations, the volume impedance distribution within the thorax model was computed for an electrode separation of 8 cm under normal (inflated lung) conditions. Figure 4 shows the volume impedance density distributions along frontal planes located 6 cm (top) and 8 cm (middle) beneath the electrode plane (i.e., thoracic surface), with volume impedance density values expressed in units of Ω/m^3^. The results indicate that the contribution is highest directly beneath the electrode array and decreases in adjacent regions. When the electrodes are positioned on the right side of the thorax, volume impedance density over the left lung is minimal. For example, with electrodes placed at the right lower level, the total thoracic transfer impedance is 15.082 Ω. Of this value, the combined contribution of both lungs is 2.566 Ω, of which 2.453 Ω arises from the right lung alone. Thus, the lungs contribute approximately 17% of the total transfer impedance, with 16.26% attributable to the right lung. Additionally, as the depth from the electrode plane increases, the spatial extent of the sensitive region expands; however, the average planar volume impedance density decreases as shown in figure 4 (bottom). This behavior reflects current spreading with depth and reduced localized sensitivity in deeper tissue layers.

**Fig.5:**
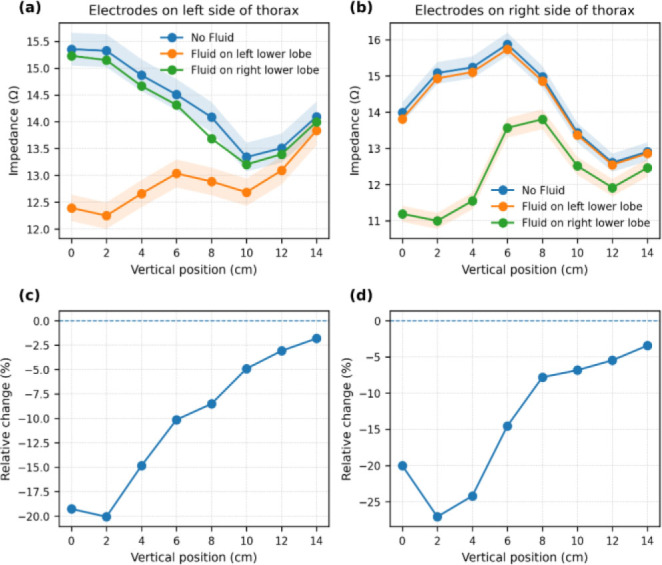
Vertical variation of transfer impedance along the thorax. (a) Left-side electrode configuration under healthy (no fluid) and fluid-accumulation conditions. (b) Right-side electrode configuration under the same conditions. (c) Relative impedance change (%ΔZ) for the left-side configuration. (d) Relative impedance change (%ΔZ) for the right-side configuration.

Variations in impedance across different vertical positions of the thorax were obtained by systematically sweeping the electrode array along both sides of the chest as shown in figure 5. Figure 5a presents the measured impedance with electrodes placed at various vertical levels on the left side of the thorax, simulating three conditions: healthy lungs, fluid accumulation (0.670 L) in the right lower lobe, and fluid in the left lower lobe. Figure 5b presents the impedance values measured on the right side of the thorax under three conditions: healthy lungs, fluid accumulation in the left lower lobe, and fluid in the right lower lobe.

In the healthy condition (no fluid, inflated lungs), the impedance values were higher at the lower vertical levels and gradually decreased at upper levels.

In the presence of fluid accumulation in the lower lobes, a consistent reduction in impedance was observed at vertical levels corresponding anatomically to the fluid-filled regions. This effect was more pronounced at positions directly above the fluid accumulation, and at higher vertical levels, the impedance difference between the fluid and non-fluid cases was reduced, reflecting the localized nature of impedance measurement sensitivity. Moreover, the impedance measured on the right side of the thorax when fluid accumulation was present in the lower lobe of the left lung was very close to the healthy condition, indicating minimal influence of fluid in the left lung on measurements from the right side and vice versa. To account for potential measurement uncertainty, a ±2% noise band was introduced around the baseline and fluid-condition curves. The observed impedance differences exceeded this tolerance range at lower vertical positions, demonstrating that the predicted changes are distinguishable from typical measurement variability.

Additionally, the relative impedance change (%ΔZ) between fluid and no-fluid conditions was computed for both left- and right-side electrode placements as shown in figure 5c and figure 5d. The percentage change profiles further highlight the spatial sensitivity of the measurement configuration, with peak relative changes occurring near the vertical levels corresponding to fluid accumulation.

**Fig.6:**
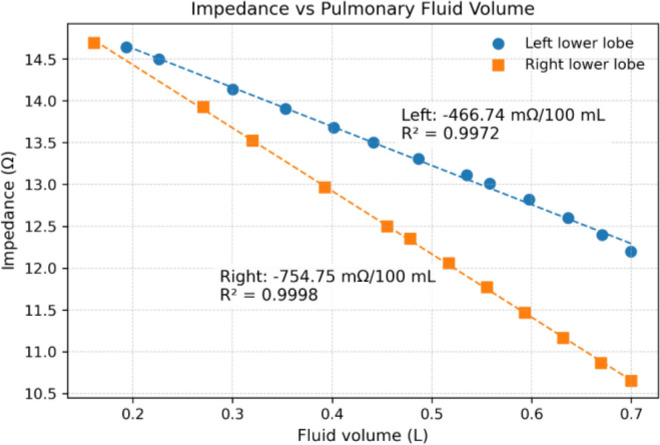
Variation of impedance for different amounts of fluid accumulation in the lower lobes of the left and right lungs, with electrodes placed on the chest surface directly above the simulated fluid buildup.

Figure 6 illustrates the variation in transfer impedance at the lower vertical electrode position on the left and right sides of the thorax as a function of simulated fluid accumulation in the lower lung lobes. A linear decrease in impedance was observed with increasing fluid volume. The coefficient of determination was r^2^ = 0.9972 for the left-side configuration and r^2^ = 0.9998 for the right-side configuration, indicating a good linear fit. This linear trend aligns with previous studies reporting a direct relationship between impedance and the volume of objects embedded within a volume conductor [26]. The slopes of the linear fitted curves were -466.74 mΩ / 100 mL and −754.75 mΩ / 100 mL on left and right side of the lungs respectively.

**Fig.7:**
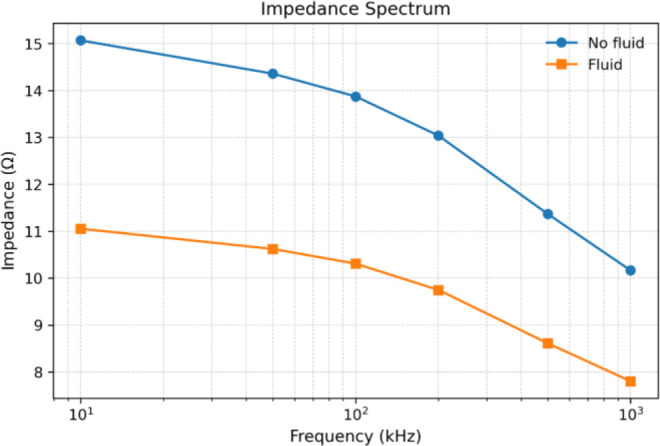
Impedance spectrum of the thorax under baseline (no fluid) and fluid-accumulation conditions.

Figure 7 shows the transfer impedance spectrum of the thorax under baseline (no fluid) and fluid-accumulation conditions from 5 to 1000 kHz. The impedance magnitude decreased with increasing frequency due to reduced capacitive reactance and frequency-dependent tissue conductivity. The relative difference between the no-fluid and fluid-accumulation conditions was observable across all frequencies, with the magnitude of impedance change varying with frequency. The impedance contrast between fluid and no-fluid conditions was slightly more pronounced at lower frequencies (5–50 kHz), suggesting increased sensitivity to extracellular conductivity changes in this range.

## Discussion

This study demonstrates the potential of electrical bioimpedance as a non-invasive and non-ionizing method for monitoring fluid accumulation in the lungs. Using a simplified finite element model (FEM) of the human thorax, based on segmented CT images, the study evaluated spatial sensitivity and impedance variations under healthy and pathological conditions. The sensitivity analysis (Figure 4) showed that the tetrapolar configuration exhibits localization beneath the electrode array, with minimal cross-thoracic influence.

In the healthy condition (no fluid, inflated lungs), the impedance values were higher at the lower vertical levels and gradually decreased at upper levels. This trend can be attributed to the anatomical distribution of lung tissue, where lung volume, and thus its contribution to the measured impedance is greater at lower levels. Moreover, at higher levels, non-lung tissues such as muscle and fat are thicker beneath the skin, further reducing the contribution of lung tissue to the measured impedance.

It can be noted from figure 5 that the relative percentage change in impedance is lower on the left side (20%) of the thorax compared to the right side (27%). This difference may be attributed to the presence of the highly conductive cardiac region, which alters current distribution in the left hemithorax.

The results show that impedance measurements are sensitive to localized fluid accumulation, particularly when electrodes are positioned directly above the affected lung region (figure 6). A consistent decrease in impedance with increasing fluid volume further suggests the potential of using impedance measurements for qualitative monitoring of lung fluid changes in disease progression. The predicted sensitivity (0.47–0.75 Ω per 100 mL) exceeds typical instrumental resolution of modern tetrapolar impedance measurement systems. For clinically relevant fluid accumulations (≥300 mL) [25], the expected impedance change would be several ohms, suggesting that such variations are practically detectable under controlled measurement conditions. As noted earlier, the lower sensitivity observed for the left-side configuration may be attributed to anatomical asymmetry and the presence of the highly conductive cardiac region in the left hemithorax.

Despite promising findings, the study is subject to several limitations. This investigation was based on computational modeling with homogeneous tissue properties assigned. The FEM model of the thorax excluded important anatomical structures such as skin, and major blood vessels. These tissues have distinct electrical properties and can significantly affect current distribution, potentially altering the accuracy of the simulations. Real-world factors such as respiratory motion, electrode–skin interface impedance, postural variation, temperature variation [27] and inter-subject anatomical variability were not included. Furthermore, fluid accumulation was modeled as a uniform conductivity change within the lower lobes, whereas clinical pulmonary edema may exhibit heterogeneous distribution. To enhance the accuracy and clinical applicability of this approach, future studies should incorporate more comprehensive and anatomically detailed FEM models, including anisotropic nature of tissues. Furthermore, modeling the dynamic behavior of the thorax during respiration would enable more physiologically realistic simulations. Future work should also include experimental validation using physical phantoms and in vivo studies, incorporation of subject-specific anatomical variability, and evaluation of robustness under physiological and measurement-related variability.

## Supplementary Material

**Table S1:** Electrical conductivity and relative permittivity values assigned to the thoracic tissue domains at different frequencies.

Tissue	Parameter	Frequency (Hz)						
		5,000	10,000	50,000	100,000	200,000	500,000	1000,000
Lungs	Conductivity σ (S/m)	0.08914	0.093172	0.10265	0.10735	0.11287	0.12301	0.13609
	Relative Permittivity ε_r_	33529	17174	4272.5	2581.3	1664.1	1025	733.18
Soft tissue	Conductivity σ (S/m)	0.18014	0.18233	0.188033	0.193132	0.204333	0.235367	0.26388
	Relative Permittivity ε_r_	27582.75	13497	5133.21	4091.043	3216.858	1840.93	931.811
Ribs	Conductivity σ (S/m)	0.051324	0.051527	0.052032	0.052342	0.052852	0.054448	0.057376
	Relative Permittivity ε_r_	1914.26	1089	438.685	349.675	295.58	241.33	196.74
Heart	Conductivity σ (S/m)	0.13665	0.15421	0.19543	0.21511	0.23824	0.28072	0.32753
	Relative Permittivity ε_r_	128090	70054	16982	9845.8	6001.1	3264.5	1967.3
Fluid	Conductivity σ (S/m)	1.5	1.5	1.5	1.5	1.5001	1.5003	1.5007
	Relative Permittivity ε_r_	98.958	98	98.558	97.99	96.556	91.35	84
Electrodes	Conductivity σ (S/m)	4.00E+06	4.00E+06	4.00E+06	4.00E+06	4.00E+06	4.00E+06	4.00E+06
	Relative Permittivity ε_r_	1	1	1	1	1	1	1
